# Gain-of-function p53^R175H^ blocks apoptosis in a precursor model of ovarian high-grade serous carcinoma

**DOI:** 10.1038/s41598-023-38609-5

**Published:** 2023-07-14

**Authors:** Jacob Haagsma, Bart Kolendowski, Adrian Buensuceso, Yudith Ramos Valdes, Gabriel E. DiMattia, Trevor G. Shepherd

**Affiliations:** 1 The Mary and John Knight Translational Ovarian Cancer Research Unit, London Regional Cancer Program, London, ON Canada; 2 Department of Anatomy and Cell Biology, Schulich School of Medicine and Dentistry, Western University, London, ON Canada; 3 Department of Oncology, Schulich School of Medicine and Dentistry, Western University, London, ON Canada; 4 Department of Biochemistry, Schulich School of Medicine and Dentistry, Western University, London, ON Canada; 5 Department of Obstetrics and Gynaecology, Schulich School of Medicine and Dentistry, Western University, London, ON Canada; 6 London Regional Cancer Program, 790 Commissioners Road East, Room A4-836, London, ON N6A 4L6 Canada

**Keywords:** Cancer, Cell biology

## Abstract

Ovarian high-grade serous carcinoma (HGSC) is a highly lethal malignancy for which early detection is a challenge and treatment of late-stage disease is ineffective. HGSC initiation involves exfoliation of fallopian tube epithelial (FTE) cells which form multicellular clusters called spheroids that colonize and invade the ovary. HGSC contains universal mutation of the tumour suppressor gene *TP53*. However, not all *TP53* mutations are the same, as specific p53 missense mutants contain gain-of-function (GOF) properties that drive tumour formation. Additionally, the role of GOF p53 in spheroid-mediated spread is poorly understood. In this study, we developed and characterized an in vitro model of HGSC based on mutation of *TP53* in mouse oviductal epithelial cells (OVE). We discovered increased bulk spheroid survival and increased anchorage-independent growth in OVE cells expressing the missense mutant p53^R175H^ compared to OVE parental and *Trp53*ko cells. Transcriptomic analysis on spheroids identified decreased apoptosis signaling due to p53^R175H^. Further assessment of the apoptosis pathway demonstrated decreased expression of intrinsic and extrinsic apoptosis signaling molecules due to *Trp53* deletion and p53^R175H^, but Caspase-3 activation was only decreased in spheroids with p53^R175H^. These results highlight this model as a useful tool for discovering early HGSC transformation mechanisms and uncover a potential anti-apoptosis GOF mechanism of p53^R175H^.

## Introduction

Epithelial ovarian cancer (EOC) is the most lethal gynaecological malignancy in the developed world, with a late stage 5-year survival rate of only 30%^1^. Due to a lack of early detection strategies, most patients are not diagnosed until the disease is already metastatic. The current standard-of-care for late stage EOC consists of debulking followed by chemotherapy, which is largely ineffective due to recurrence of drug-resistant disease^2^. As such, improved screening and more effective therapies are required for improving patient outcomes.

The most common and most lethal histotype of EOC is ovarian high-grade serous carcinoma (HGSC). The origin of HGSC was long thought to be the ovarian surface epithelium, as the primary tumour is most often found at the ovary. However, recent findings have challenged this notion and uncovered the distal fallopian tube as a major source of HGSC^3–6^. The first known molecular alteration of normal fallopian tube epithelium is mutation of the tumour suppressor gene *TP53*, an event considered universal in HGSC^7,8^. *TP53* mutation induces a precursor phenotype called the p53 signature, containing a loss of ciliated cells, and an outgrowth of secretory cells with DNA damage accumulation^5^. p53 signatures progress to serous tubal intraepithelial carcinoma (STIC) lesions with increased proliferation and stratification of the epithelium^4^. Invasive HGSC is thought to occur when STIC lesion cells exfoliate from the fallopian tube as multicellular clusters called spheroids, which form primary tumours at the ovary and secondary tumours throughout the peritoneal cavity^9,10^.

Compared to the hematological route of metastasis observed in many cancers, spheroid-mediated dissemination of HGSC occurs more efficiently, without the need for crossing barriers during intravasation and extravasation. Spheroids provide resistance to anoikis, a form of apoptosis induced by loss of cellular attachment^11^, and contribute to disease recurrence by remaining dormant in the ascites of patients following surgery providing resistance to proliferation-targeting chemotherapies^12^. The central role of spheroids in HGSC initiation and metastasis presents challenges for developing successful treatment strategies, but may also represent vulnerabilities. Understanding the mechanisms upon which spheroids depend will help uncover novel biomarkers and therapeutic targets. However, more complex models that accurately recapitulate the unique progression pattern of HGSC are required.

As *TP53* mutation is an early and universal event, targeting mechanisms driven by this alteration is an attractive approach. The role of *TP53* mutation in cancer has traditionally been attributed to loss of its tumour suppressive functions. However, the majority of *TP53* mutations are missense mutations within the DNA binding domain, resulting in increased stability, dominant-negative activity, and gain-of-function (GOF) properties through aberrant transcriptional regulation and protein binding interactions^13^. In the context of HGSC, both missense mutation and deletion of *TP53* occur, but missense mutations are more common^14^. GOF p53 mutants have been associated with invasion, chemoresistance, and worsened patient survival^15–18^, but the significance of GOF p53 in HGSC initiation is poorly understood. Mechanistically, the outcome of GOF p53 mutants in cancer can be dependent on the specific point mutation present, as p53 missense mutations occur at hotspot residues within the DNA-binding domain that can uniquely alter the protein- and DNA-binding capabilities of p53^13,19^. In HGSC, some of these mechanisms are just beginning to be understood. p53^R248W^ and p53^R273H^ increased BDNF/TrkB signaling through increased TrkB recycling, resulting in enhanced migration of HGSC cell lines and increased metastasis in mice^20^. In the same study, p53^R175H^ produced the same phenotype through a distinct mechanism by directly increasing *TrkB* transcription. p53^R248Q^ and p53^S215R^ formed a complex with ERβ2 which bound to the *FOXM1* promoter to increase its transcription, resulting in increased proliferation and carboplatin resistance on HGSC cell lines^18^. In a human fallopian tube epithelium model, p53^R175H^ increased mesothelial clearance and matrix production through increased transcription of *TWIST1*^21^. In the present study, we develop and characterize an in vitro model suitable for assessing GOF mechanisms driven by mutant p53 in HGSC precursor cells. By engineering mouse oviductal epithelial (OVE) cells, we identified a transformed phenotype due to expression of the missense mutant p53R175H, but not deletion of *Trp53*. Additionally, we uncover a potential anti-apoptotic GOF property of p53R175H in OVE spheroids.

## Results

### p53 missense mutation enhances OVE growth in vitro

**Figure 1:**
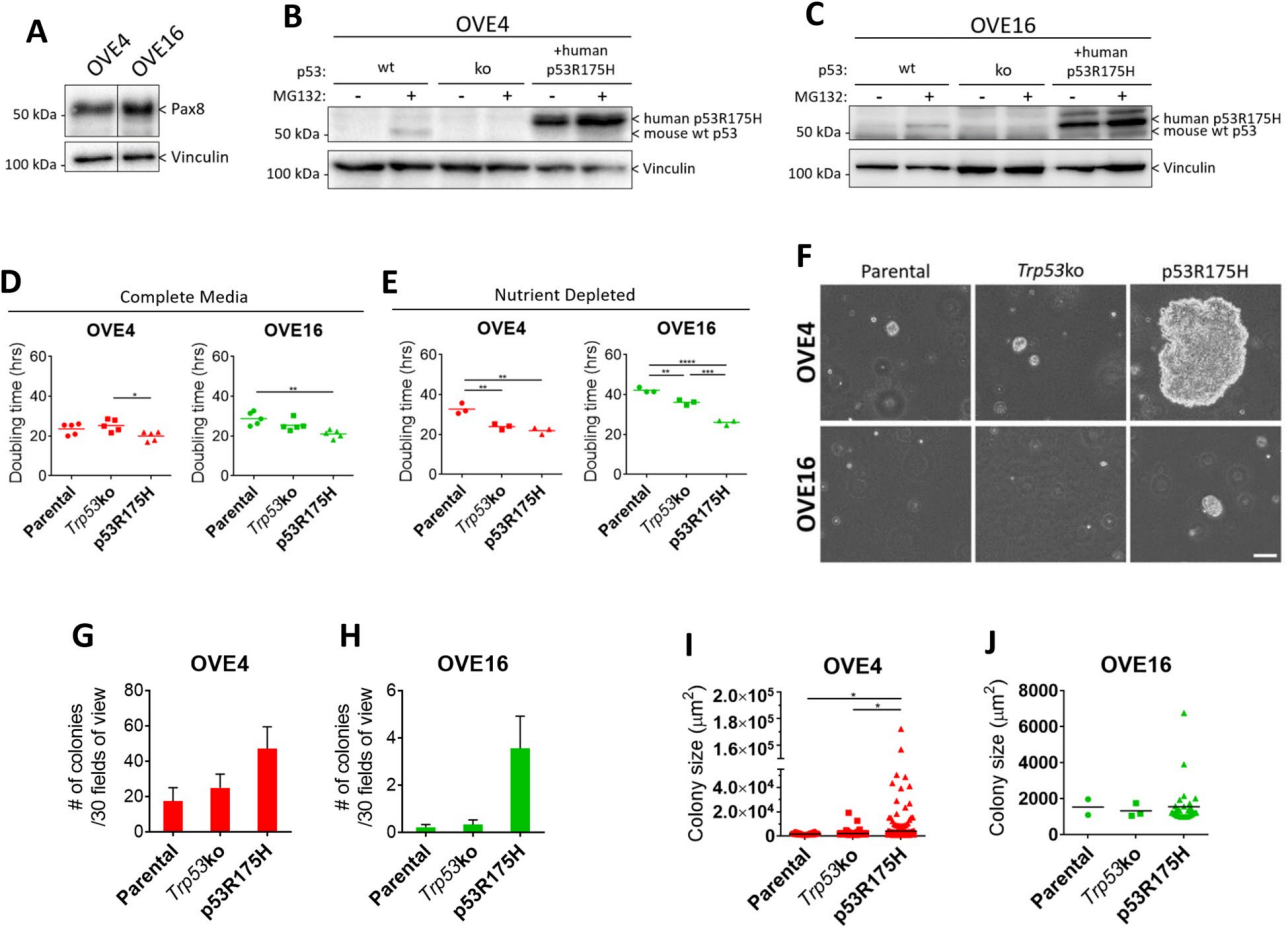
p53^R175H^ produces a transformed phenotype in OVE cells. (**A**) Immunoblot analysis of Pax8 in parental OVE cells. Vinculin was used as a loading control. (**B**,**C**) Immunoblot analysis of p53 in OVE cell lines. To detect endogenous p53, adherent OVE cells were treated with 10 µM of the proteasome inhibitor MG132, or vehicle control, for 6 h. Vinculin was used as a loading control. Doubling times were calculated for adherent cells in (**D**) complete media or (**E**) AMEM + 0.1% FBS using confluency-over-time data generated in the Incucyte ZOOM live cell analysis system. (**F**) Phase-contrast images of soft agar colonies at 21 days. Scale bar represents 100 µm. Colony formation was quantified by measuring (**G**,**H**) colony number and (**I**,**J**) colony size from images of 30 random fields of view per well. All statistical analyses were performed using one-way ANOVA followed by Tukey’s multiple comparisons test (*p < 0.05; **p < 0.01; ***p < 0.001; ****p < 0.0001; n = 3–5). Error bars represent standard error of the mean. Original blots are presented in Supplemental Fig. S5.

To generate a model of precursor HGSC cells, we used the previously described OVE4 and OVE16 cell populations that were independently isolated from the oviducts of female FVB/n mice^22^. To confirm the origin of these lines, expression of the secretory oviductal epithelial cell marker Pax8 was assessed by immunoblot (Fig. 1A). In both OVE cell lines, *Trp53* was stably knocked out by CRISPR-Cas9 genome editing, and the human p53 missense mutant p53^R175H^ was stably expressed by lentiviral transduction as verified by immunoblotting (Fig. 1B,C). We confirmed that the *Trp53* locus was effectively targeted by CRISPR/Cas9 via PCR followed by Sanger sequencing of genomic DNA isolated from the OVE4- and OVE16-*Trp53*ko lines as compared with the respective parental cell lines. OVE16-*Trp53*ko cells contained the expected 70-bp deletion generated by the two sgRNA sequences. OVE4-*Trp53*ko cells were heterozygous having both the same deletion as well as a single nucleotide insertion in the other allele resulting in a frameshift (Supplemental Fig. S1). Aberrant proliferation is a prominent feature of HGSC precursor lesions. p53 signatures form from secretory cell outgrowth, and epithelial stratification arises in STICs^4,5^. To assess if *Trp53* mutations increase proliferative potential in OVE cells, doubling time was calculated. When cultured in complete media, OVE4 and OVE16 monolayer cells expressing p53^R175H^ exhibit modest decreases in doubling time compared to OVE4-*Trp53*ko and OVE16 parental cells, respectively (Fig. 1D). When cultured in nutrient-depleted conditions, the differences in growth between OVE cell lines were amplified, demonstrated by an increase in doubling time in parental OVE cells compared to OVE cells with *Trp53* deletion or expression of p53^R175H^ (Fig. 1E).

To further assess the transformed state of OVE cells, we measured colony formation in soft agar. The total number of colonies > 1000 µm^2^ was unchanged due to *Trp53* deletion or expression of p53^R175H^ (Fig. 1F–H). However, p53^R175H^ enabled a drastic increase in colony growth in a small subset of colonies, demonstrated by in increase in colony size in OVE4-p53^R175H^ compared to OVE4 and OVE4-*Trp53*ko cells (Fig. 1F,I). OVE16 and OVE16-*Trp53*ko cells possessed minimal colony formation with only 2 and 3 colonies meeting the size threshold, respectively, while OVE16-p53^R175H^ cells demonstrated a trend towards increased colony size (Fig. 1F,J). Overall, p53^R175H^ expression is promoting a more transformed in vitro phenotype in OVE cells.

### p53^R175H^ enhances OVE spheroid growth and viability

**Figure 2:**
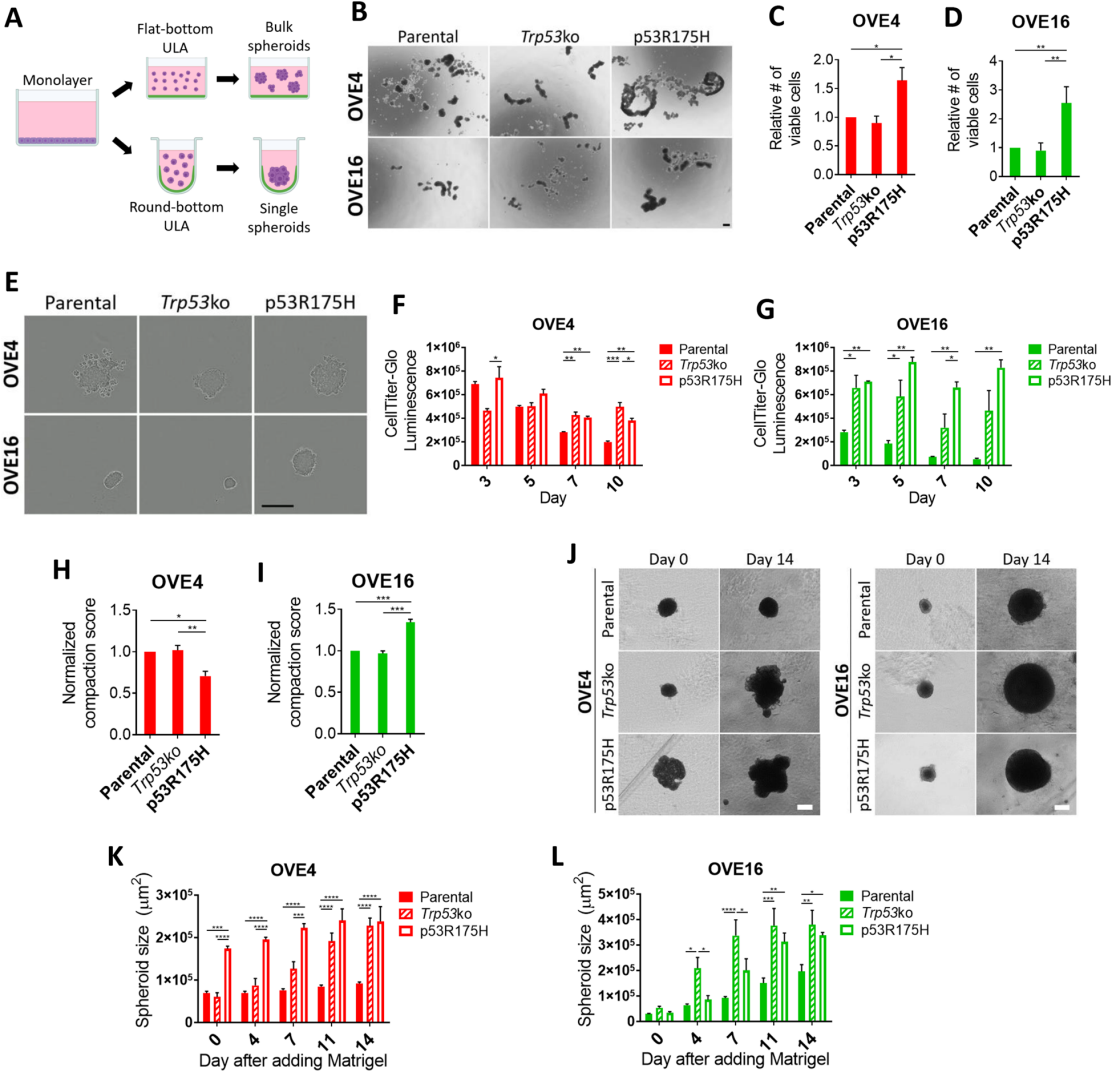
*Trp53* mutation increases spheroid viability and growth. (**A**) Schematic of spheroid culture. (**B**) Images of bulk OVE spheroids at day 3. Scale bar represents 200 µm. (**C**,**D**) Trypan Blue exclusion cell counting on bulk OVE spheroids at 72 h. (**E**) Images of single OVE spheroids at 72 h. Scale bar represents 300 µm. (**F**,**G**) CellTitre glo viability on single OVE spheroids at day 3, 5, 7, and 10. (**H**,**I**). Compaction scores of single OVE spheroids at day 3. (**J**) Images of single OVE spheroids at day 0 and 14 after embedding in Matrigel. Scale bars represent 200 µm. (**K**,**L**) Single OVE spheroid size at day 0, 4, 7, 11, and 14 after embedding in Matrigel. All statistical analyses were performed using one-way ANOVA followed by Tukey’s multiple comparisons test (*p < 0.05; **p < 0.01; ***p < 0.001; ****p < 0.0001; n = 3–4). Error bars represent standard error of the mean.

In order to colonize the ovary, STIC lesion cells must survive following detachment from the ovary. To recapitulate this event in our system, we seed single cells in flat-bottom or round bottom Ultra-Low Attachment (ULA) plates to generate bulk spheroids or single uniform spheroids, respectively (Fig. 2A). When cultured in flat-bottom ULA plates, OVE cells spontaneously form spheroids with varying size and morphology based on p53 status, with expression of p53^R175H^ producing the largest spheroids for OVE4 and OVE16 cells (Fig. 2B). In agreement with this, OVE4-p53^R175H^ and OVE16-p53^R175H^ bulk spheroids possess increased viable cells at day 3 compared to parental and *Trp53*ko counterparts (Fig. 2C,D).

When cultured in round-bottom ULA plates, OVE4 cells with either *Trp53* mutation formed smoother, more uniform spheroids compared to the parental line, which exhibits cells on the periphery that do not incorporate into the spheroids (Fig. 2E). In OVE16 cells, p53^R175H^ expression produces much larger spheroids compared to parental and *Trp53*ko cells. Using the luminescence-based CellTiter-Glo viability assay, cell survival of single spheroids was measured over time. OVE4 cells had similar viability regardless of p53 status at day 5, but by day 10 spheroids with either *Trp53* mutation had increased viability compared to parental cells (Fig. 2F). In OVE16 cells, p53^R175H^ expression increased viability compared to parental cells across all time points. OVE16-*Trp53*ko spheroids had comparable viability to OVE16-p53^R175H^ at day 3, but failed to maintain this viability at day 7 (Fig. 2G). Further interpretation of this data implies that cells within OVE16-p53^R175H^ spheroids may be proliferating, as the relative number of viable cells increased over time.

Based on the morphology of spheroids formed in round-bottom ULA plates, it appears as though parental OVE4 spheroids are less compact compared with spheroids possessing p53 mutations. To address this phenotype directly, spheroid compaction was calculated by measuring viability of day-3 single spheroids and dividing by spheroid size. Surprisingly, OVE4-p53^R175H^ spheroids had a decreased compaction score compared to OVE4 and OVE4-*Trp53*ko spheroids (Fig. 2H). With respect to spheroid cell viability, OVE4 and OVE16 spheroids with p53^R175H^ expression produced similar phenotypes (Fig. 2C,D,F,G). However, OVE16-p53^R175H^ spheroids had increased compaction compared to parental and *Trp53*ko spheroids (Fig. 2I), representing an opposite phenotype to that of OVE4 spheroids. These results suggest the ability to form compact spheroids is not required for maintaining viability.

Upon surviving detachment from the fallopian tube, spheroids must adhere to the ovary and invade the underlying stroma to form a primary tumour. To assess the ability of OVE spheroids to invade and grow in an extracellular matrix, pre-formed single spheroids were embedded in Matrigel and spheroid size was measured over 2 weeks. Spheroids formed by OVE4-p53^R175^ had increased size compared to parental and *Trp53*ko spheroids already at the time of adding Matrigel (Fig. 2J,K). While OVE4-p53^R175H^ spheroids displayed modest growth, OVE4-*Trp53*ko spheroids increased in size nearly fourfold by day 14. Parental OVE4 spheroids had limited growth. In OVE16 spheroids, both *Trp53* deletion and expression of p53^R175H^ increased spheroid size at day 14 compared to parental spheroids, with *Trp53* deletion displaying rapid growth as early as day 4 (Fig. 2J,L). Interestingly, in addition to increased spheroid growth in Matrigel, OVE spheroids with *Trp53* deletion or expression of p53^R175H^ demonstrated a qualitative increase in clonogenicity. Upon addition of Matrigel to the spheroids, some of the cells at the periphery of the spheroid detached as single cells embedded in Matrigel. Parental cells that exfoliated from the spheroid arrested or died off, while either *Trp53* mutation enabled some of these cells to form new colonies. This phenomenon occurred in only a subset of spheroids, but was exclusive to spheroids with *Trp53* mutations (Supplemental Fig. S2). Overall, *Trp53* deletion and p53 missense mutation provide unique pro-tumorigenic spheroid phenotypes, with both types of mutations enhancing properties relevant to multiple steps in early HGSC development.

### p53 mutations alter OVE sensitivity to carboplatin

**Figure 3:**
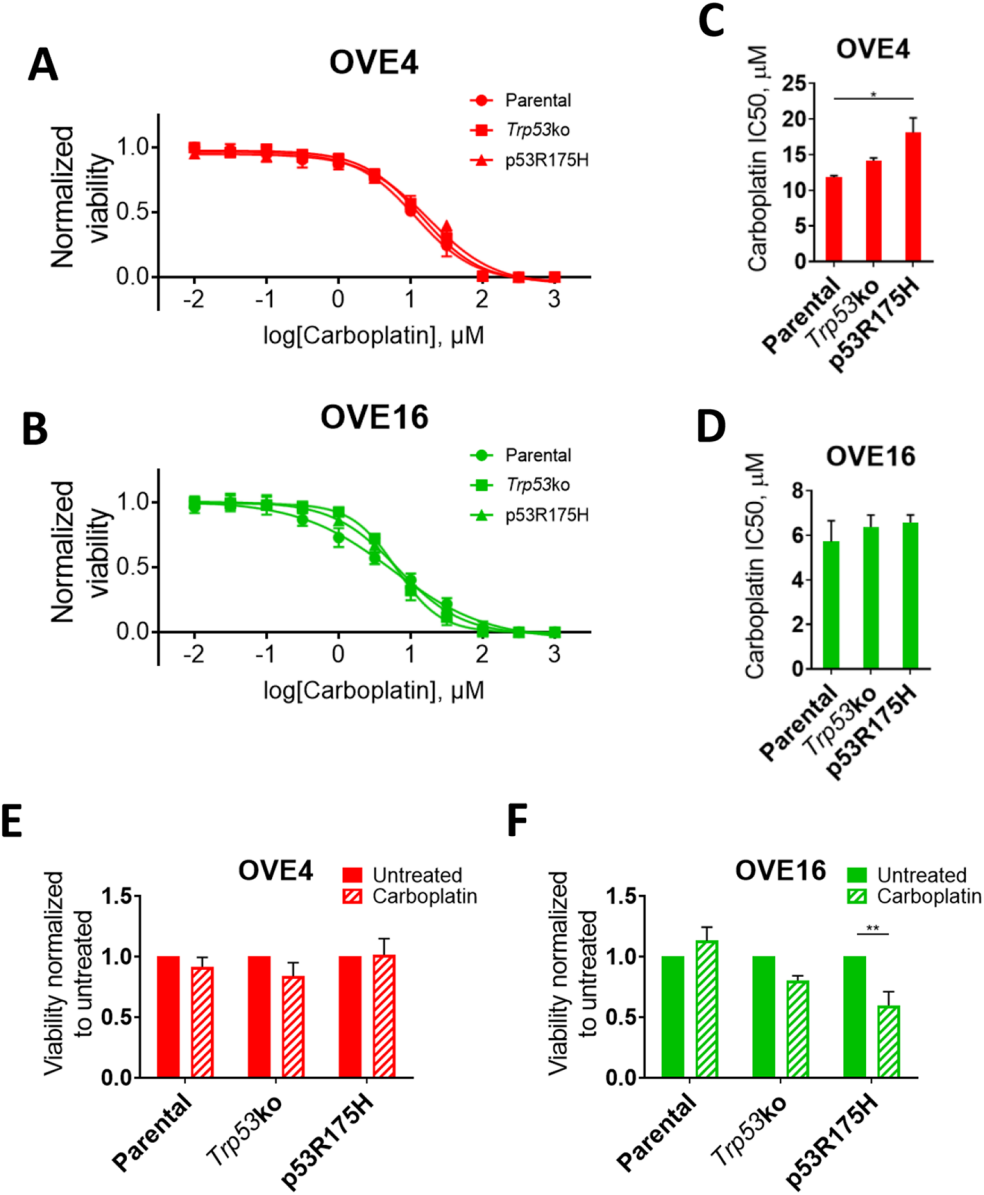
OVE cell carboplatin sensitivity. (**A**,**B**) Carboplatin dose–response curves and (**C**,**D**) IC50 values for monolayer OVE cells determined by alamarBlue cell viability assay. Statistical analyses were performed using one-way ANOVA followed by Tukey’s multiple comparisons test (*p < 0.05; n = 3). (**E**,**F**) Bulk OVE spheroid sensitivity to carboplatin relative to untreated controls determined by alamarBlue cell viability assay. Spheroids were treated with the monolayer IC50 value of their respective parental OVE cell line. Statistical analyses were performed using two-way ANOVA followed by Šidák’s multiple comparisons test (**p < 0.01; n = 3). Error bars represent standard error of the mean.

To determine if p53 mutations desensitize OVE cells to a commonly used HGSC therapeutic, carboplatin dose–response curves were generated for adherent OVE4 and OVE16 cell lines (Fig. 3A,B). OVE16 lines had a similar IC50 regardless of p53 status, while OVE4 cells expressing p53^R175H^ had decreased sensitivity compared to parental OVE4 cells (Fig. 3C,D). Next, OVE4 and OVE16 bulk spheroids were treated with their respective parental adherent carboplatin IC50, and spheroid cell viability was measured. Interestingly, spheroids formed by all 3 OVE4 lines showed no difference in viability between treated and untreated spheroids, despite altered sensitivity due to p53^R175H^ in adherent culture (Fig. 3E). In contrast, OVE16-p53^R175H^ spheroids treated with carboplatin had decreased viability compared to untreated spheroids (Fig. 3F). This is consistent with the proliferative phenotype observed in Fig. 2G, as carboplatin targets proliferating cells. Parental and *Trp53*ko OVE16 cells showed no difference in viability between treated and untreated spheroids.

### Parental OVE spheroids have enrichment of apoptosis and immune-related pathways

**Figure 4:**
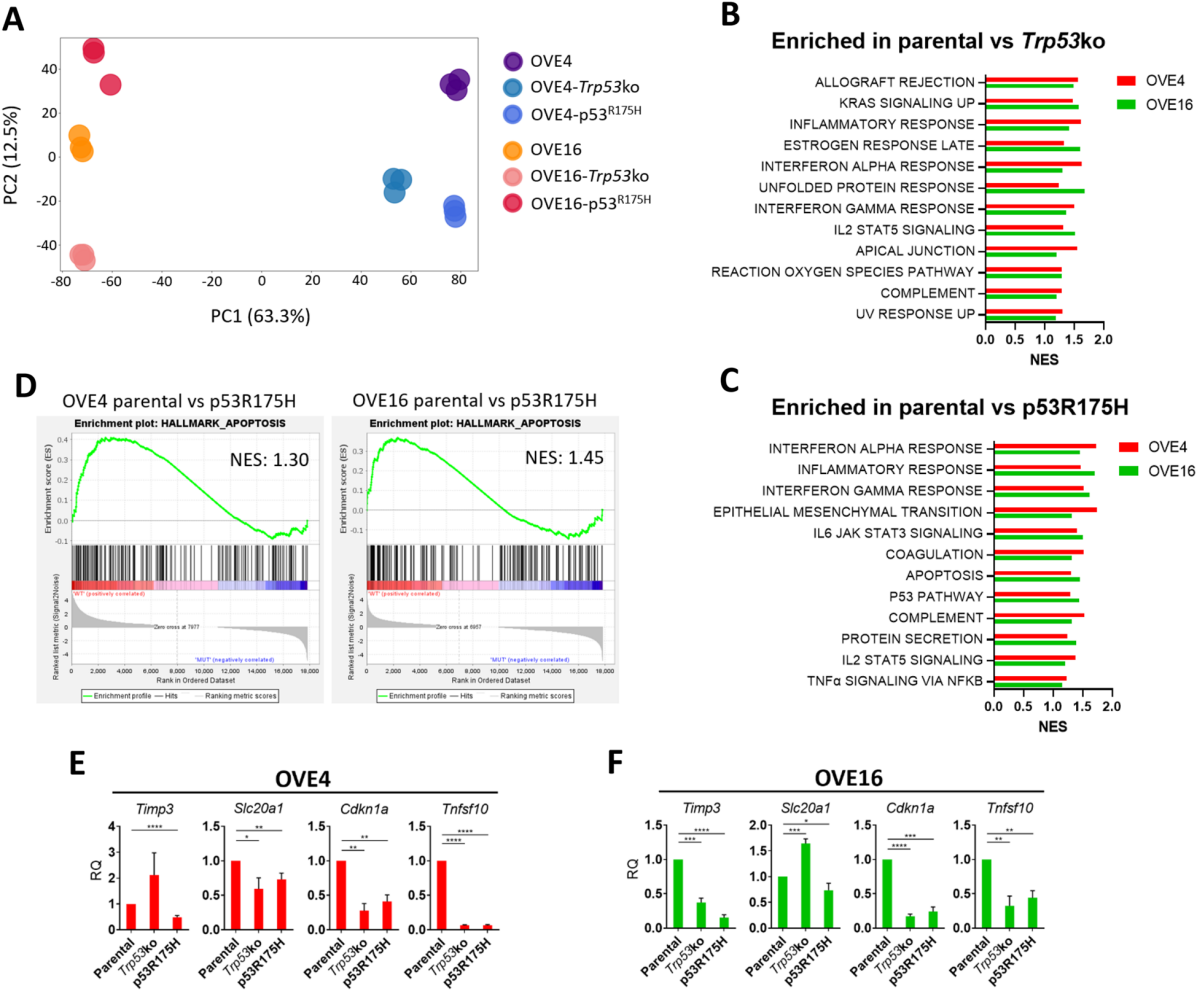
The apoptosis hallmark is enriched in parental OVE spheroids. (**A**) Principal component analysis on gene expression data from each technical replicate of each OVE cell line. Normalized enrichment scores (NES) for the top 12 gene sets in the Molecular Signatures Database Hallmarks collection that are enriched in parental OVE spheroids compared to OVE spheroids with (**B**) *Trp53* deletion or (**C**) p53^R175H^ expression (FDR < 0.25). (**D**) Enrichment plots for the apoptosis hallmark in the parental vs p53^R175H^ comparisons. (**E**,**F**) RT-qPCR validation of genes that are driving enrichment of the apoptosis hallmark in parental OVE spheroids compared to spheroids with p53^R175H^ expression. Gene expression in OVE spheroids with *Trp53* deletion or p53^R175H^ expression was compared to parental spheroid controls by unpaired, two-tailed Student’s *t*-test (*p < 0.05; **p < 0.01; ***p < 0.001; ****p < 0.0001; n = 3). Error bars represent standard error of the mean.

To assess transcriptional dysregulation due to p53 mutation, we performed RNA sequencing on total RNA isolated from OVE spheroids. Principal component analysis (PCA) demonstrated tight clustering of technical replicates, validating the quality of the RNA sequencing data (Fig. 4A). Principal component 1, which accounts for 63.3% of variance in gene expression, clusters the samples based on their parental origin. Principal component 2 clusters samples based on the status of p53, but only accounts for 12.5% of variance. These data indicate that OVE4 and OVE16 cell lines with either p53 mutation are more similar to their parental cell line than they are to their respective OVE counterpart with the same p53 mutation.

To identify pathways altered by p53 mutation that may be driving observed transformation and spheroid phenotypes, we performed gene set enrichment analysis (GSEA). More specifically, we used the Hallmarks gene sets from the Molecular Signatures Database to identify p53-dependent alterations in well-defined gene signatures that are common to both OVE4 and OVE16 spheroids. Parental OVE spheroids displayed 12 gene sets enriched compared to *Trp53*ko spheroids (Fig. 4B) and 12 gene sets enriched compared to p53^R175H^ spheroids (Fig. 4C). Interestingly, several immune-related gene sets were enriched in parental spheroids compared to spheroids with *Trp53* deletion or expression of p53^R175H^ (Supplemental Fig. S3).

We decided to pursue the apoptosis hallmark further, as it was down in spheroids expressing p53^R175H^ but not down due to *Trp53* deletion, indicative of a potential GOF of p53^R175H^. Additionally, a reduced capacity to induce apoptosis may explain the observed increase in spheroid viability due to p53^R175H^, as a form of apoptosis called anoikis is activated by loss of cellular attachment^23^. OVE4 and OVE16 parental spheroids had normalized enrichment scores of 1.30 and 1.45, respectively, compared to p53^R175H^ counterparts for the apoptosis hallmark (Fig. 4D). To validate the GSEA analysis, transcript expression was measured for genes that were driving enrichment of the apoptosis hallmark in parental spheroids compared to spheroids expressing p53^R175H^ (Fig. 4E,F). Spheroids expressing p53^R175H^ consistently had reduced expression of apoptosis-related genes compared to parental spheroids, while deletion of *Trp53* resulted in variable expression including some genes with increased expression.

### p53 is required for the intrinsic and extrinsic apoptosis pathway in OVE spheroids

**Figure 5:**
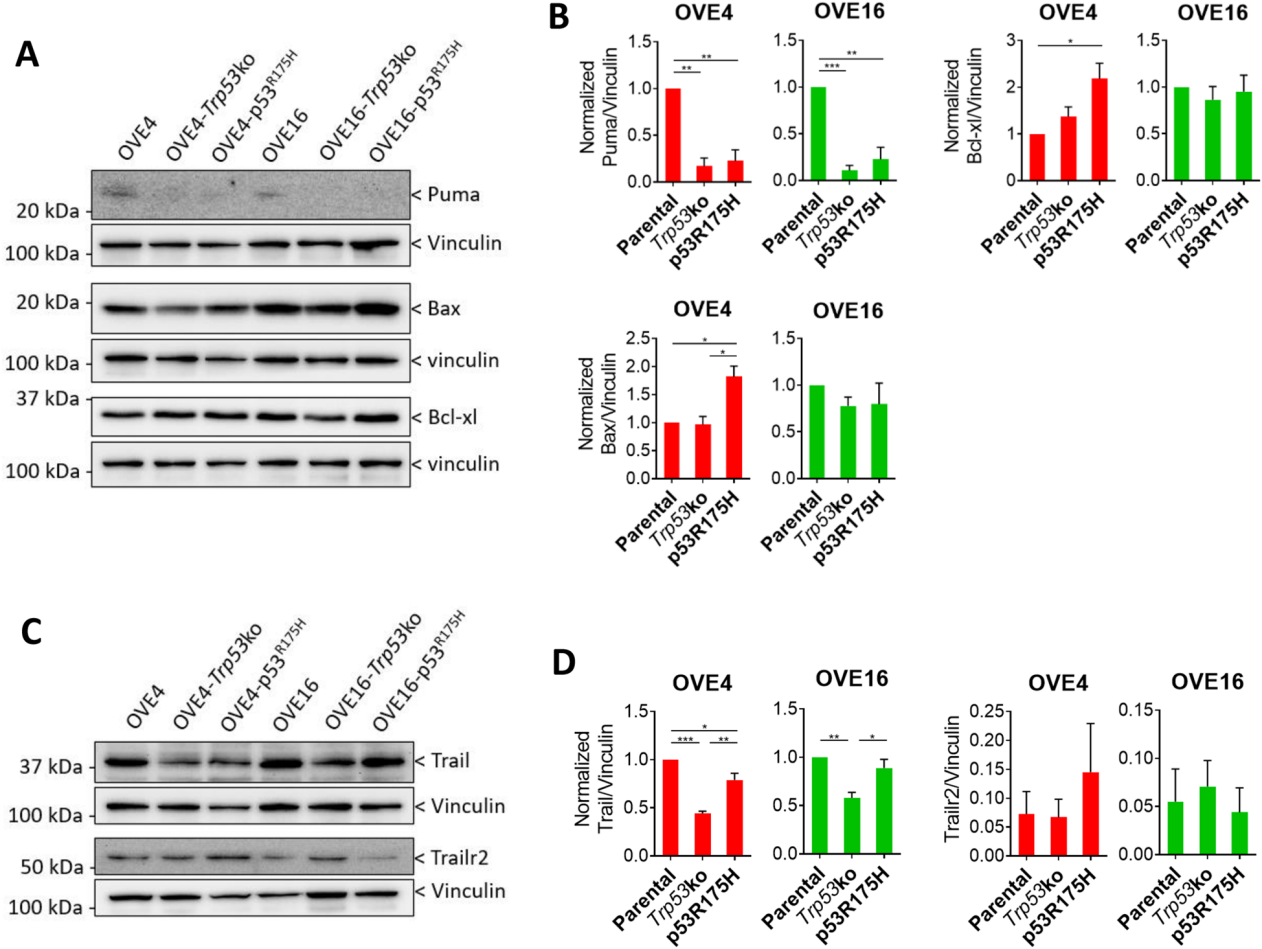
Decreased intrinsic and extrinsic apoptosis signaling in OVE spheroids with *Trp53* mutation. (**A**) Representative immunoblots and (**B**) densitometric analysis for Puma, Bax, and Bcl-xl in OVE spheroids. Vinculin was used as a loading control. (**C**) Representative immunoblots and (**D**) densitometric analysis for Trail and Trailr2 in OVE spheroids. Vinculin was used as a loading control. All statistical analyses were performed using one-way ANOVA followed by Tukey’s multiple comparisons test (*p < 0.05; **p < 0.01; ***p < 0.001; n = 3). Error bars represent standard error of the mean. Original blots are presented in Supplemental Fig. S5.

Apoptosis can be activated intrinsically through mitochondrial outer membrane permeabilization (MOMP) or extrinsically through the binding of extracellular or cell membrane-bound ligands to death receptors^24–26^. To determine whether the intrinsic or extrinsic apoptosis pathway is contributing to the observed apoptosis phenotype, we assessed expression of proteins involved in each pathway. In the intrinsic pathway, MOMP is dependent on the relative abundance of two families of outer mitochondrial membrane proteins. Pro-survival Bcl-2 family proteins antagonize MOMP, while pro-apoptosis Bax family proteins promote MOMP. A third family of pro-apoptosis BH3-only proteins can bind to and sequester pro-survival Bcl-2 family proteins to increase the relative abundance of Bax family proteins. Based on transcript expression from the RNA-seq analysis, we looked at one example of each of the families of proteins involved in the intrinsic pathway (Fig. 5A,B). The BH3-only member Puma was increased in parental OVE4 and OVE16 spheroids compared to spheroids with *Trp53* deletion or expression of p53^R175H^. With respect to Bax family member Bax and Bcl-2 family member Bcl-xl, there was no difference among OVE16 spheroids, while OVE4-p53^R175H^ had increased expression of both compared to parental OVE4 spheroids. In the extrinsic pathway, expression of the Trail ligand was decreased due to *Trp53* deletion and expression of p53^R175H^ in OVE4 spheroids, with OVE4-*Trp53*ko spheroids displaying the lowest expression (Fig. 5C,D). In OVE16 spheroids, deletion of *Trp53* resulted in decreased Trail expression compared to OVE16 and OVE16-p53^R175H^ spheroids. Importantly, all OVE spheroids expressed the Trail receptor Trailr2.

**Figure 6:**
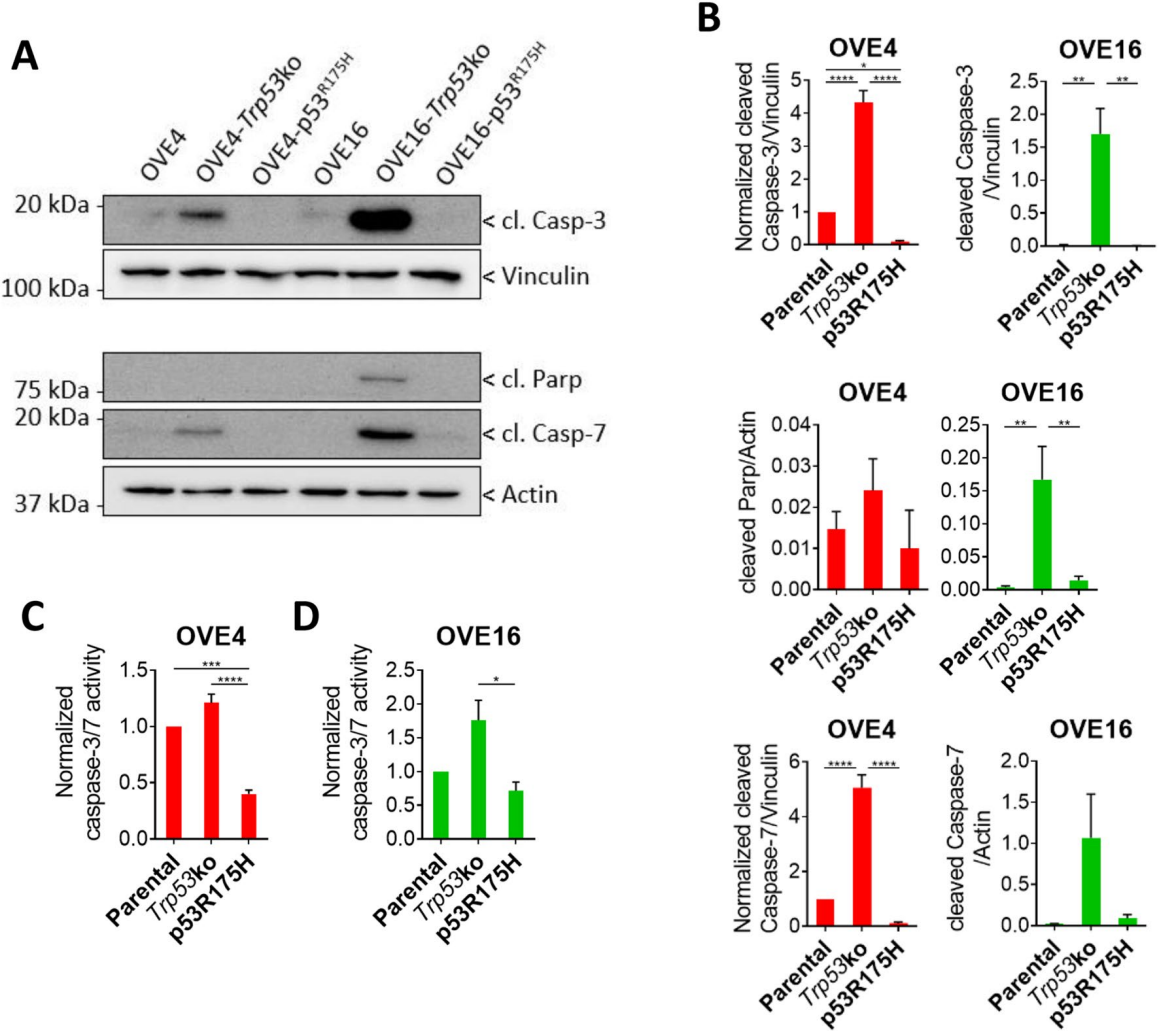
Decreased apoptosis activation in OVE spheroids with p53^R175H^ expression. (**A**) Representative immunoblots and (**B**) densitometric analysis of cleaved Caspase-3, cleaved Caspase-7, and cleaved Parp in OVE spheroids. Actin and vinculin were used as loading controls. (**C**,**D**) Caspase-3 and Caspase-7 activity in OVE spheroids determined by Caspase-Glo 3/7 assay. All statistical analyses were performed using one-way ANOVA followed by Tukey’s multiple comparisons test (*p < 0.05; **p < 0.01; ***p < 0.001; ****p < 0.0001; n = 3). Error bars represent standard error of the mean. Original blots are presented in Supplemental Fig. S5.

The intrinsic and extrinsic pathway converge on cleavage and activation of Caspase-3 and Caspase-7, and subsequent cleavage of Parp. To assess if altered upstream intrinsic and extrinsic apoptosis signaling is influencing cleavage of downstream substrates, we measured their cleaved products (Fig. 6A,B). In OVE4 spheroids, expression p53^R175H^ produced the lowest cleavage of Caspase-3, while *Trp53* deletion resulted in increased cleavage compared to parental spheroids. OVE16 and OVE16-p53^R175H^ spheroids had lower Caspase-3 cleavage compared to OVE16-*Trp53*ko. Caspase-7 cleavage demonstrated similar trends to Caspase-3, with *Trp53* deletion showing increased cleavage. Parp cleavage was similar among OVE4 spheroids, while *Trp53* deletion produced the highest cleavage among OVE16 spheroids. To validate that increased caspase cleavage is facilitating increased caspase activity, we measured cleavage of the caspase consensus sequence DEVD in OVE spheroids. OVE4-p53^R175H^ spheroids had reduced caspase activity compared to OVE4 parental and OVE4-*Trp53*ko spheroids (Fig. 6C). In OVE16 spheroids, expression of p53^R175H^ decreased caspase activity compared to deletion of *Trp53* (Fig. 6D). Given that apoptosis is being induced in spheroids with *Trp53* deletion, despite increased survival compared to parental spheroids (Fig. 1B–G), we assessed whether *Trp53* deletion yielded spheroids with a hollow core due to this cell death. Staining using H&E on individual spheroid sections demonstrated that spheroids with *Trp53* deletion did not lack cells within the core, but rather all spheroids among lines had a similar morphology (Supplemental Fig. S4). Overall, expression of p53^R175H^ is blocking apoptosis to a higher degree than *Trp53* deletion.

## Discussion

The development of mouse models that recapitulate the transformation events throughout the progression of HGSC is crucial to uncovering new therapeutic targets and biomarkers of early disease. The first known alteration leading to HGSC is mutation of *TP53* in secretory cells of the distal fallopian tube^4^. By culturing spheroids from OVE cells with *Trp53* mutations, we have characterized an in vitro precursor model with which to study some of the earliest steps in HGSC development. Importantly, direct comparison with OVE cells containing wild-type p53 provides a well-controlled system with which to elucidate the role of p53 mutations in early disease progression. Many human cell line models require immortalization of fallopian tube epithelial (FTE) cells by inactivating p53, precluding experiments with a true normal cell control for HGSC precursor cells. When culturing mouse cells it is important to maintain them at low culture as spontaneous immortalization has been observed, often through loss of downstream p53 function^27,28^. Importantly, our transcriptomic analysis identified enrichment of p53 signaling in parental spheroids compared to spheroids expressing p53^R175H^ (Fig. 4C), and we observed decreased transcript expression of the canonical p53 target *Cdkn1a* in spheroids with *Trp53* deletion or expression of p53^R175H^ (Fig. 4E,F), validating this model as an appropriate system with which to study p53 mutations. The transition from p53 signatures to STIC lesions involves the acquisition of a proliferative phenotype^5^. We observed a modest increase in proliferative potential due to *Trp53* mutation in OVE cells, in support of this system as a STIC lesion model. Apart from *TP53* mutation, there are no high-frequency somatic point mutations in STIC lesions, rather it is characterized by high copy number variations^29^. Loss of *TP53* function has been associated with increase genomic stability in FTE cells^30,31^, and further evidence suggest an enhanced role of p53^R175H^ in promoting genomic instability in a mouse model of breast cancer^32^. However, studies on the copy number status of OVE cells with altered p53 are needed to conclude whether this model reflects the genomic instability observed in human STIC.

To increase the relevance of our OVE model further, we cultured them as spheroids in suspension culture to replicate the detachment of STIC lesion clusters from the fallopian tube. Additionally, spheroids facilitate metastasis by detaching from the primary ovarian tumour and passively spreading throughout the peritoneal cavity^9^. While this mode of metastasis eliminates the need for invasion in and out of the bloodstream or lymphatic vessels, providing an easier route for cancer cells, it also provides potential vulnerabilities through the targeting of spheroid survival mechanisms. The OVE model discussed in this study lacks manipulation of driver genes involved in advanced HGSC, such as loss of *NF1*, loss of *PTEN*, or *CCNE1* amplification (TCGA). As such, our current cell lines are better suited to study early disease compared to metastasis. However, advancements in genome editing technologies potentiates further modifying OVE cells to uncover mechanisms driven by other genetic alterations that are enhancing spheroid-mediated spread. Additionally, some mechanisms of spheroid survival may be common to both advanced and early disease, such as dormancy. Studies on spheroids derived from epithelial ovarian cancer patient ascites have demonstrated that spheroids enter a dormant state, providing resistance to chemotherapy^33^. A similar phenotype may be occurring in our OVE model, as small differences in sensitivity of the OVE4 cell lines to carboplatin in adherent culture are abrogated in spheroid culture, where spheroids treated with carboplatin have similar viability to untreated spheroids. However, these precursor cells likely have a lower proliferative potential compared to HGSC cells and would naturally be less sensitive to proliferation-targeting chemotherapies.

High patient heterogeneity observed in HGSC supports a precision medicine approach to treating this disease^8,34^. However, early universal mutation of *TP53* suggests that mechanisms driven by this alteration in the fallopian tube epithelium may be common across patient populations^7,8^. In this study, we used two independently isolated OVE cell populations to account for inherent differences between individuals. Indeed, PCA on RNA-seq data demonstrated that OVE4 and OVE16 cell lines with *Trp53* mutations are more similar to their respective parental cell line than they are to the independent OVE cell line with the same mutation. This is not surprising, given that the number of genes not regulated by p53 far outweighs the number of p53-regulated genes. Parental OVE4 and OVE16 cell lines had different capacities to form colonies in soft agar; OVE4 cells were in fact capable of forming small colonies whereas OVE16 cells failed to do so, as expected for non-transformed cells (Fig. 1F–J). These differences could be explained by a recent study highlighting distinct transcriptional profiles of secretory epithelial cells from different regions of the oviduct^35^. Alternatively, the OVE4 and OVE16 cell lines may have diverged over time in culture, with OVE4 gaining additional growth advantages. However, this specific phenotypic difference between OVE4 and OVE16 lines did not preclude our objective of assessing the overall effect of *Trp53* mutations in OVE cell transformation. Our downstream analysis of transcript profiles focused on the common overlap in biological pathways between OVE4 and OVE16 cell lines, which yielded consistent validation in our subsequent analysis of apoptosis dysregulation. To identify signaling pathways that are altered by *Trp53* mutations in OVE cells independent of heterogenous backgrounds, our downstream analysis focused the pathway overlap between OVE4 and OVE16 cells lines. Importantly, we identified several pathways enriched in both parental OVE cell lines compared to their *Trp53* mutation-containing derivatives.

A key part of a cells defense against cancer is the ability to enter apoptosis following loss of detachment from the extracellular matrix. This type of apoptosis, called anoikis, is particularly important in HGSC prevention. Unlike most other cancers which present the primary tumour at the site of origin, HGSC initiation requires the survival of FTE cells following detachment from the fallopian tube basement membrane. Previous studies in FTE cells demonstrated resistance to anoikis due to several p53 missense mutants compared to cells deleted for *TP53*^21^. In agreement with this, OVE spheroids expressing p53^R175H^ had increased viability compared to both parental spheroids and *Trp53*ko spheroids, accompanied by a downregulation of the apoptosis hallmark and reduced Caspase-3 and Caspase-7 activity in p53^R175H^ spheroids. OVE spheroids with *Trp53*ko had a survival advantage over parental spheroids in some contexts, but Caspase-3 and Caspase-7 activity was similar between the two groups, or even higher in the *Trp53*ko spheroids. These data support the notion that *Trp53* deletion is not analogous to p53 missense mutation. In the context of apoptosis, this drastic difference in Caspase cleavage and activity driven by the two types of p53 mutations is interesting, given that several pro-apoptotic genes involved in the early signaling events of the intrinsic and extrinsic apoptosis pathway are transcriptional targets of p53^36–38^. p53 also post-translationally inactivates pro-survival Bcl-2 family members^39^. Indeed we observed decreased upstream pro-apoptosis signaling due to both *Trp53* deletion and p53^R175H^. The divergent results of upstream apoptosis signaling and Caspase cleavage and activity may point to a GOF mechanism of p53^R175H^ at the level of Caspase activity, which has previously been observed in bone, lung, and colon cancer cell lines^40,41^. Alternatively, p53^R175H^ may contain GOF properties at the level of upstream apoptosis signaling member transcription through inactivation of p53 family members p63 and p73, which are also involved in transcriptional activation of apoptosis genes^42–44^. p53 missense mutants, including p53^R175H^, can bind to and inactivate p63 and p73 transcriptional activity, representing an anti-apoptosis mechanism precluded by *Trp53* deletion^45–49^. While the upstream apoptosis signaling members assessed in this study demonstrated similar expression due to both *Trp53* mutations, additional members of the Bax, Bcl-2, and BH3-only families, as well as additional extrinsic pathway ligands and receptors may be differentially expressed due to p53^R175H^ GOF mechanisms. Regardless of the mechanism responsible, this work has uncovered differential antagonism of the apoptosis pathway by *Trp53* deletion and p53^R175H^.

The interaction between cancer cells and stromal or immune cells within HGSC tumours plays a key role in the progression of the disease. As just two examples, the presence of CD3+ tumour-infiltrating T cells has been correlated with an improved 5-year survival rate^50^, and mesenchymal stem cell derived IL-6 secretion increases ovarian cancel cell invasion^51^. Our transcriptomic analysis uncovered several different inflammatory- and immune-related hallmarks that are enriched in parental OVE spheroids compared to *Trp53*ko and p53^R175H^-expressing spheroids. Downregulated inflammatory signaling could represent an immune evasion mechanism that is dependent on *Trp53* mutations. While the biological significance of these pathways was not assessed in our in vitro model, our thorough characterization in this study, and the observed inflammatory phenotype provides strong support for expanding this model to an in vivo setting. Importantly, the use of mouse cells enables these studies to be performed in immune-competent, syngeneic mice, providing an accurate representation of the tumour microenvironment with which to assess the pro- or anti-tumorigenic role of altered inflammatory signaling. In conclusion, we have characterized a novel in vitro model of early HGSC, and provided proof-of-principle of its ability to uncover signaling pathways.

## Methods

### Cell culture

The OVE4 and OVE16 cell lines were provided by Dr. Barbara Vanderhyden (University of Ottawa, Ottawa, ON) and have been previously described^22^. All OVE cell lines were cultured in AMEM (Winsent CAT# 310-022-CL) supplemented with 5% FBS (Wisent CAT# 098150), MEM non-essential amino acids (Gibco CAT# 11140-050), 0.01 mg/mL insulin-transferrin-selenium solution (Sigma-Aldrich CAT# 11074547001), 0.01 µM estradiol (Sigma-Aldrich CAT# E2257), and 2 ng/mL human EGF (Sigma-Aldrich CAT# E9644). Adherent cultures were grown on tissue culture-treated polystyrene plates (Sarstedt). Spheroid cultures were maintained on Ultra-Low Attachment (ULA) plates (Corning).

### Generation of *Trp53*ko and p53^R175H^ OVE cell lines

For *Trp53* deletion, two independent pSpCas9-sgTrp53 plasmids were generated as previously described^52^, using the *Trp53*-targeting sequences 5′-AGTGA AGCCC TCCGA GTGTCagg-3′ (site 1) and 5′-AACAG ATCGT CCATG CAGTGagg-3′ (site 2). OVE cells at 80% confluency were transfected with 1.5 µg of each pSpCas9-sgTrp53 plasmid using Lipofectamine LTX and PLUS reagents (Invitrogen) according to the manufacturer’s instructions. Transfection media was removed after 8 h and replaced with complete media. The next day, cells were treated with media containing 2 µg/mL puromycin and treated for one day. After cells recovered, limiting dilution was performed to grow up colonies from single *Trp53*-knockout cells. Colonies were expanded for protein collection, and loss of *Trp53* was confirmed by immunoblotting. Five colonies were combined at equal numbers to generate pooled populations.

For generating lines stably expressing p53^R175H^, lentiviral particles containing the p53^R175H^ expression construct were generated. HEK293T cells at 60–70% confluency were transfected with a pLenti6/V5-p53_R175H plasmid (Addgene Plasmid #22936), and viral vectors containing psPAX2 (Addgene Plasmid #12260) and pMD2.G (Addgene Plasmid #12259) at a ratio of 10:6:4 using Lipofectamine 2000 (Invitrogen CAT# 11668019) according to the manufacturer’s instructions. After cells expressed the virus for 48 h, media was collected and filtered through a 0.45 micron filter, combined with 8 µg/mL polybrene and added to the OVE cells. After 24 h, media was removed and replaced with fresh media containing blasticidin to select for cells expressing the target vector. After 7 days of selection, cells were expanded for use.

### PCR and sequencing

DNA was isolated using the Wizard Genomic DNA Purification Kit (Promega CAT#A1120). The *Trp53* gene was amplified at exon 3 using primers designed to flank the two CRISPR target sites (Supplemental Fig. S1A). PCR products were run on a 1% agarose gel with the RedSafe nucleic acid stain (Intron Biotechnology CAT#21141) and imaged using the ChemiDoc Imaging System (Bio-Rad). For sequencing, OVE4-*Trp53*ko PCR products were isolated using the QIAEX II Gel Extraction Kit (Qiagen CAT#20021). PCR products for all other cell lines were purified using the QIAquick PCR Purification Kit (Qiagen CAT#28104). PCR products were combined with the forward primer or reverse primer and sent to the London Regional Genomics Centre at Robarts Research Institute for Sanger sequencing.

### Preparation of whole cell lysates

For assessing Pax8, adherent cells at 80% confluency were washed with PBS and scraped into modified RIPA buffer containing 50 mM HEPES (pH 7.4), 150 mM NaCl, 10% glycerol, 1.5 mM MgCl_2_ 1 mM EGTA, 1% Triton X-100, 0.1% SDS, 1 mM Na_3_VO_4_, 10 mM NaF, 1 mM PMSF, 1 × SIGMA*FAST* protease inhibitor cocktail (Sigma-Aldrich CAT# S8820). Cells were lysed on ice for 30 min with vortexing every 5 min. Lysates were centrifuged (21,100*g*, 4 °C, 20 min), and protein was collected from the supernatant.

For assessing p53, adherent cells at 80% confluency were treated with 10 µM of the proteasome inhibitor MG132 (Sigma-Aldrich CAT# C2211) or vehicle control for 6 h. After treatment, cells were lysed and protein collected as described above.

For assessing apoptosis signaling members in spheroids, 500,000 OVE4 cells, or 1,000,000 OVE16 cells in 3 mL media were seeded in 6-well ULA plates. After 24 h, spheroids were pelleted and washed with PBS. Modified RIPA buffer was added to the pellets, and cells were lysed and protein collected as described above.

### Immunoblot analysis

Proteins were resolved by SDS-PAGE on 8, 10, or 12% gels. Proteins were transferred to PDVF membranes (Roche CAT# 03010040001) at 100 V for 1 h. Non-specific binding was blocked with 5% milk or bovine serum albumin in tris-buffered saline + Tween 20 for 1 h. Primary antibodies were diluted in blocking buffer and incubated overnight at 4 °C. Membranes were incubated with peroxidase-conjugated anti-mouse or anti-rabbit IgG antibodies for 1 h, then exposed to chemiluminescent reagent. Membranes were imaged using the ChemiDoc Imaging System (Bio-Rad) and densitometry was performed using Image Lab 6.1 software package (Bio-Rad).

### Antibodies and reagents

Antibodies against Puma (CAT# 14570; 1:1000), Bax (CAT# 14796; 1:1000), Bcl-xl (CAT# 2762; 1:1000), cleaved Caspase-3 (CAT# 9661; 1:500), cleaved Caspase-7 (CAT# 9491; 1:500), and cleaved Parp (CAT# 9541; 1:1000) were purchased from Cell Signaling Technology. Antibody against Pax8 (CAT# 10336-1-AP; 1:20,000) was purchased from Proteintech. Antibody against p53 (CAT# OP03; 1:1000) was purchased from Calbiochem. Antibodies against Trail (CAT# NB500-220; 1:1000) and Trailr2 (CAT# NB100-56618; 1:1000) were purchased from Novus Bio. Antibodies against actin (CAT# A2066; 1:20,000) and vinculin (CAT# V9264; 1:50,000) were purchased from Sigma-Aldrich. HRP-conjugated antibodies against rabbit IgG (CAT# NA934; 1:10,000) and mouse IgG (CAT# NA931; 1:10,000) were purchased from Cytiva. Antibodies were diluted in tris-buffered saline-Tween 20 containing 5% non-fat milk or 5% bovine serum albumin.

### Doubling time

7500 cells in 1 mL media were seeded in 48-well tissue culture-treated polystyrene plates (Corning CAT# 3548). Images were captured at 2-h intervals in the Incucyte ZOOM live cell analysis system (Sartorius), and the masking feature was used to measure confluency over time. Growth curves and doubling time calculations were generated in GraphPad Prism 9. To measure doubling time in nutrient depleted conditions, cells were seeded in complete media and allowed to adhere for 24 h, after which complete media was replaced with AMEM + 0.1% FBS.

### Growth in soft agar

1.5 mL of media + 0.5% agar (Bio Basic CAT# D0012) was added to a 6-well plate. After the agar solidified, 1 mL of media + 0.5% agar containing 50,000 cells was added on top. After the agar containing cells solidified, 2 mL of media was added on top. Media was replaced with fresh media every week. After 21 days, 30 images of random fields of view were captured. Colonies > 1000 µm^2^ in size were measured using the Trainable Weka Segmentation plugin^53^ in the *Fiji Is Just ImageJ* (Fiji) image analysis software^54^.

### Spheroid viability

For bulk spheroid viability, 100,000 cells in 1.4 mL media were seeded in a 24-well flat-bottom ULA plate. After 3 days, spheroids were collected and washed twice with PBS. Spheroids were trypsinized in 50 µL trypsin for 30 min with pipetting every 10 min to dissociate to single cells. Trypsin was inactivated with 50 µL FBS, and viable and total cells were counted with Trypan Blue exclusion counting and a TC20 cell counter (BioRad). For single spheroid viability over time, 2000 cells in 100 µL media were seeded in a 96-well round bottom ULA plate. At day 3, 5, 7, and 10, cell viability was determined by Cell-Titer-Glo luminescent cell viability assay (Promega).

### Spheroid compaction

2000 cells in 100 µL media were seeded in a 96-well round bottom ULA to generate single spheroids. At day 3, images were captured on an Olympus CKX53 inverted microscope and the relative amount of viable cells was determined by Cell-Titer-Glo luminescent cell viability assay. Spheroid size was measured with *Fiji* as described above. Spheroid compaction score was determined by dividing the relative number of viable cells by spheroids area.

### Spheroid invasion in Matrigel

Spheroid invasion was assessed using a modified protocol from Vinci et al.^55^. Briefly, 4000 cells in 100 µL were seeded in a 96-well round-bottom ULA to generate single spheroids. After 3 days, 50 µL of media was removed and 50 µL of Matrigel (Corning) was added. After the matrix solidified, 100 µL media was added on top. Images were at day 0, 4, 7, 11, and 14 after adding Matrigel. Spheroid size was measured with *Fiji* as described above.

### Carboplatin treatments

To determine monolayer carboplatin IC50 values, 1000 cells in 100 µL media were seeded in a 96-well plate. The next day, cells were treated with a serial dilution of carboplatin ranging from 10 nM to 1 mM. After 72 h of treatment, cell viability was determined using the alamarBlue Cell Viability Reagent (Invitrogen CAT# DAL 1025) according to the manufacturer’s instructions, and IC50 values were calculated using GraphPad Prism 9.

To assess spheroid sensitivity to carboplatin, 10,000 cells in 100 µL media were seeded in a 96-well flat-bottom ULA plate to generate bulk spheroids. After 72 h, spheroids were treated with their respective monolayer carboplatin IC50 values for an additional 72 h. Following treatment, viability was determined by alamarBlue viability assay.

### RNA isolation

500,000 cells in 3 mL we seeded in a 6-well flat-bottom ULA plate to generate bulk spheroids. After 72 h, spheroids were pelleted and washed with PBS, and RNA was isolated using the RNEasy Spin Column Kit (Qiagen CAT# 74104) according to the manufacturer’s instructions. Genomic DNA was removed by DNAseI (Qiagen CAT# 79254) treatment. RNA concentration, A_260/280_, and A_260/230_ were measured on a spectrophotometer.

### Transcriptomic analysis

For RNA-seq analysis, isolated RNA from OVE spheroids was collected in triplicate and sent to the London Regional Genomics Centre and Robarts Research Institute for library preparation. RNA quality was first confirmed using Agilent 2100 Bioanalyzer sequencing and sequencing was performed on an Illumina NextSeq using 75-bp single end reads. Raw sequencing reads were uploaded to the Galaxy platform at https://usegalaxy.org/^56^ and tested for quality using FastQC to measure the per base sequence quality, GC content, N content, overrepresented sequences and adapter content. Reads were mapped to the mouse genome (mm10, Genome Reference Consortium Mouse Build 38) using HISAT2^57^ (Galaxy Version 2.2.1 + galaxy1) and read counts at annotated genomic features were obtained using featureCounts^58^ (Galaxy Version 2.0.1 + galaxy2). Differential expression was determined using the DESeq2^59^ package with standard settings (Galaxy Version 2.11.40.7 + galaxy2). Pathway analysis was performed by gene set enrichment analysis (GSEA; Broad Institute)^60^ using the Hallmarks gene set collection from the Molecular Signatures Database.

### RT-qPCR

cDNA was generated from total RNA using the High Capacity cDNA Reverse Transcriptase Kit (ThermoFisher CAT# 4368814) according to the manufacturer’s instructions, using 1000 ng RNA per reaction, with a final volume of 20 µL per reaction. Reactions were incubated in a MyCycler thermocycler (BioRad) with the following cycles: 25 °C for 10 min, 37 °C for 120 min, 85 °C for 5 min, 4 °C until sample retrieval. qPCR was performed using the Brilliant II SYBR Green Master Mix (Agilent Technologies CAT# 600828) according to the manufacturer’s instructions with a 10 µL reaction volume. Cycling was performed in a QuantStudio 3 RT-PCR system (ThermoFisher). Data analysis was performed using the QuantStudio Design and Analysis Software v1.1.0, using the 2^−ΔΔCT^ method to calculate fold-change relative to controls. Primers (Supplementary Table S1) were purchased from Invitrogen.

### Caspase-Glo assay

10,000 cells in 100 µL media were seeded in a 96-well flat-bottom ULA to generate bulk spheroids. After 24 h, 100 μL of the Caspase-Glo 3/7 reagent (Promega CAT# G8091) was added to each well. Plates were incubated in the dark for 30 min on a plate rocker followed by 30 min with no rocking. Wells were transferred to a 96-well opaque white plate and luminescence was read on a Biotek plate reader. To normalize caspase activity to the relative number of viable cells, spheroid viability was determined in parallel by quantifying the relative amount of DNA using the CyQUANT Cell Proliferation Assay (ThermoFisher CAT# C7026). Spheroids were pelleted (4 °C, 3 min, 500*g*) and media was aspirated. Pellets were frozen at − 80 °C for 10 min to help lysis. 200 µL of CyQUANT buffer/dye solution was added to each tube followed by vortexing. After an additional freeze thaw cycle, samples were transferred to a 96-well opaque white plate and fluorescence was read on a Biotek plate reader.

### Histology

Single spheroids were generated in 96-well round-bottom ULA plates. At day 3, spheroids were collected, washed twice with PBS, and fixed in 10% formalin for 15 min. Spheroid pellet was resuspended in 3% agarose, polymerized at 4 °C overnight, then placed into cassettes and processed through graded alcohols to paraffin. Pellet was embedded, 5 µm sections were cut onto charged slides, and H&E staining was performed.

### Statistics

Statistical analyses were performed using GraphPad Prism 9.4.1. Specific details are provided in figure legends.

## Supplementary Information

Supplementary Information.

## Data Availability

RNA-seq data is available at the NCBI GEO Accession GSE227681.
