# Supplementary material for: Gain-of-function p53R175H blocks apoptosis in a precursor model of ovarian high-grade serous carcinoma
Source: Sci Rep. 2023 Jul 14;13:11424. doi: 10.1038/s41598-023-38609-5 (PMC10349050; doi:10.1038/s41598-023-38609-5)
Supplement: Supplementary file 1 — Supplementary Information. [file 41598_2023_38609_MOESM1_ESM.docx]

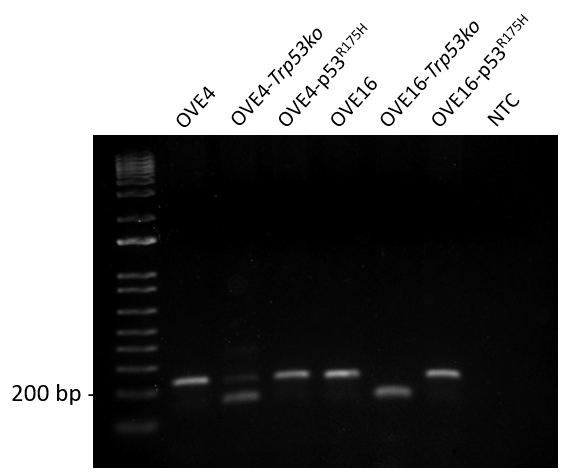

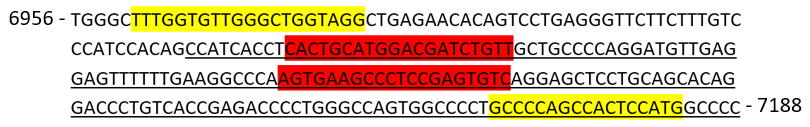


**B**

**A**

**C**


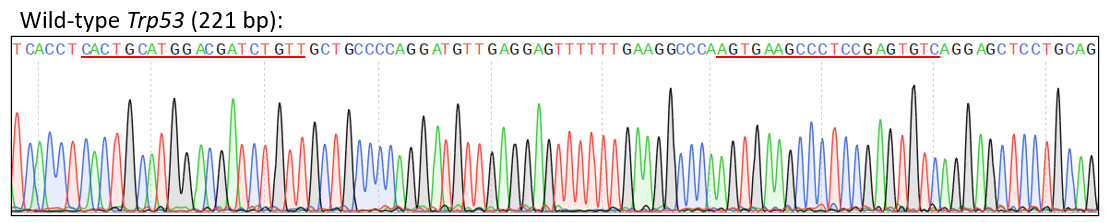


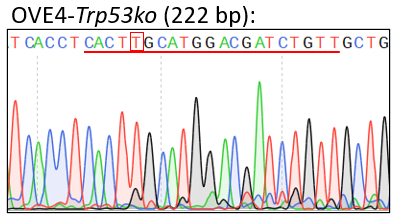

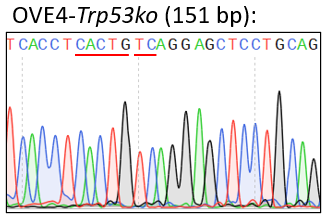

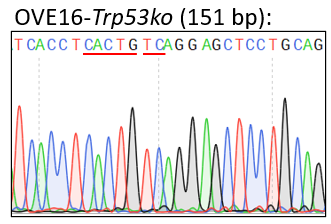


**D**

**E**

**F**

**G**


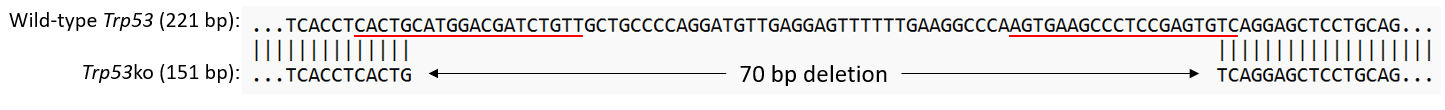


**H**


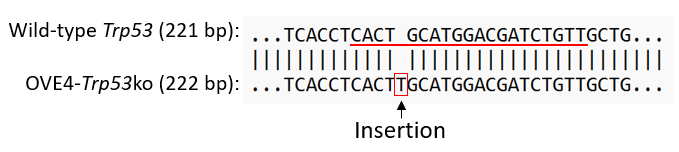


**Supplemental Figure S1:** Confirmation of *Trp53* deletion in OVE cells. **A**) Primer sequences (yellow) and CRISPR target sequences (red) in the *Trp53* gene. Underlined sequence represents the start of exon 3. **B**) PCR products from amplification of genomic DNA from each OVE cell line. NTC: No template control. Electropherograms from Sanger sequencing of **C**) parental OVE4 and OVE16 PCR products and **D-F**) OVE4-*Trp53*ko and OVE16-*Trp53*ko PCR products. **G,H**) Sequence alignment between parental OVE lines and OVE-*Trp53*ko lines. Red underlines represent CRISPR target sequences.


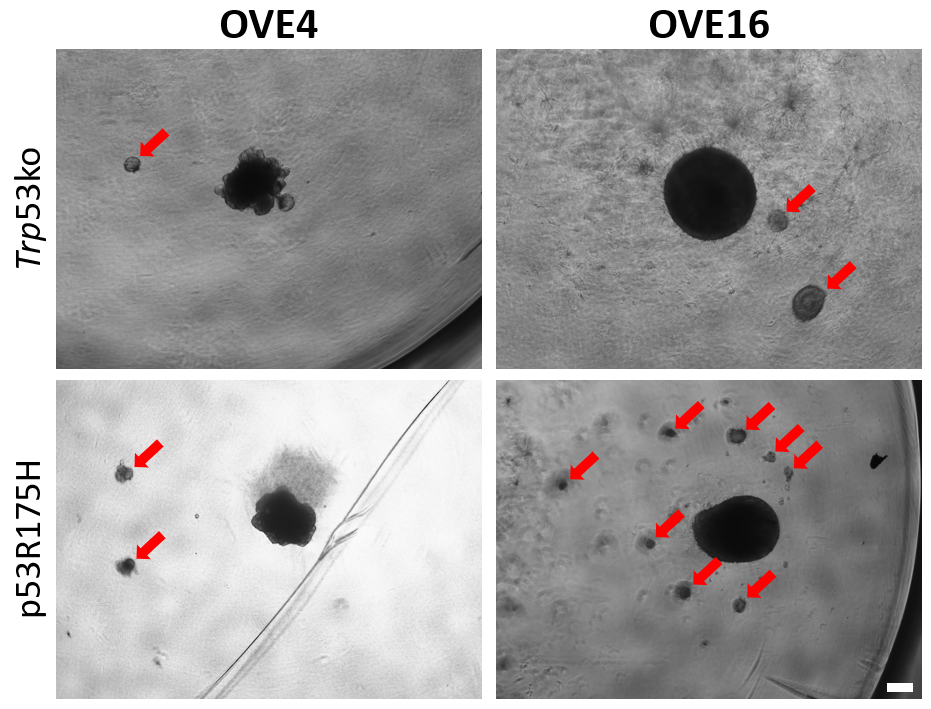


**Supplemental Figure S2:** Images of OVE spheroids with *Trp53* mutation 14 days after embedding in Matrigel. Red arrows indicate satellite colonies. Scale bar represents 200 µm.


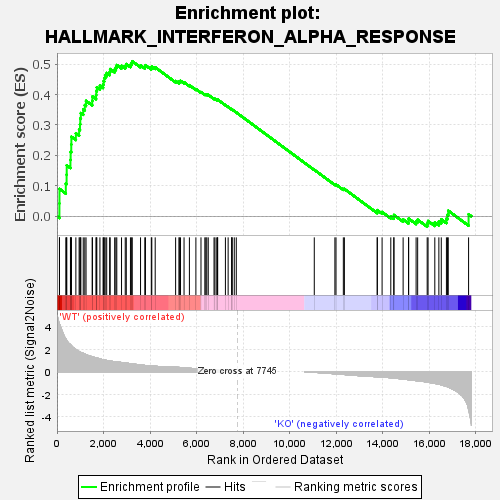

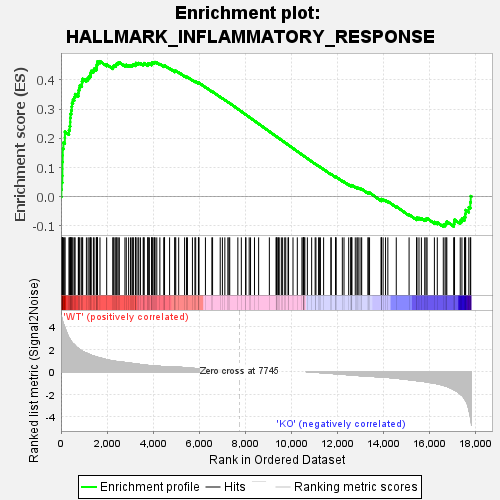

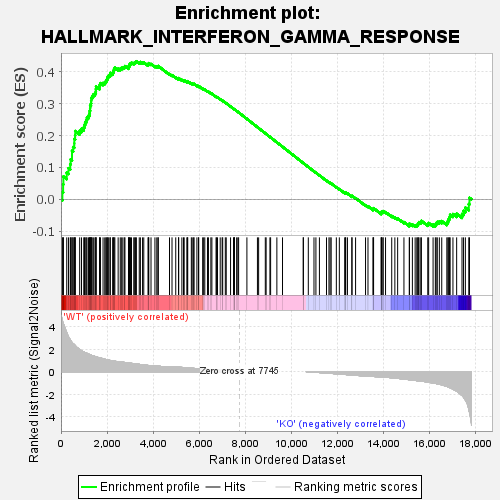

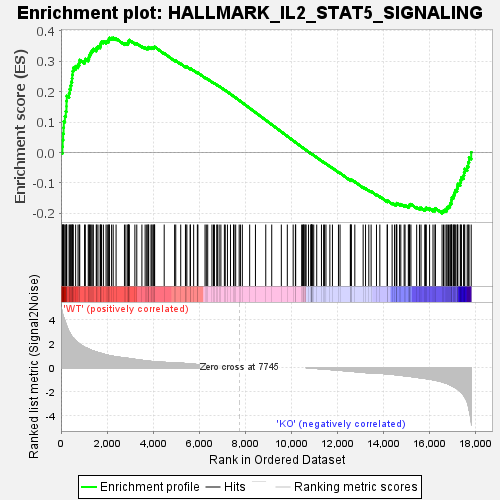


OVE4 parental vs *Trp53*ko:

OVE16 parental vs *Trp53*ko:


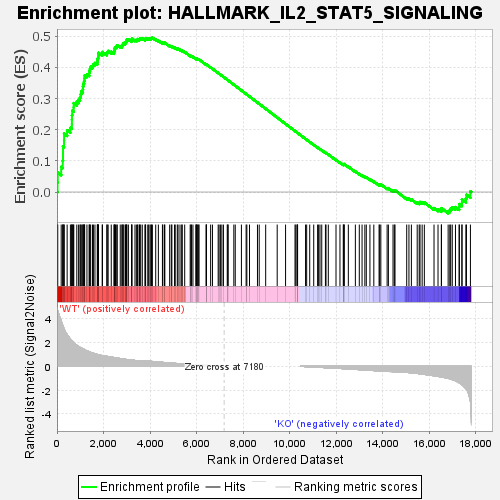

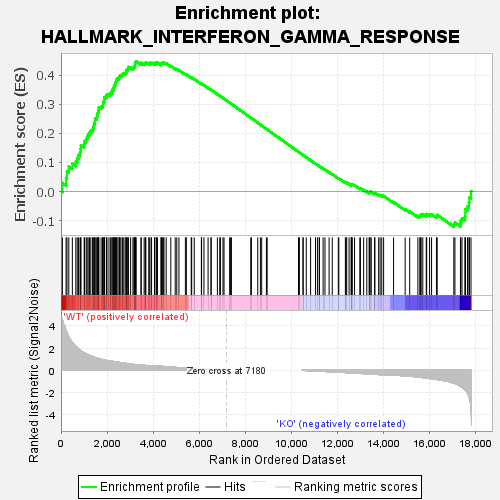

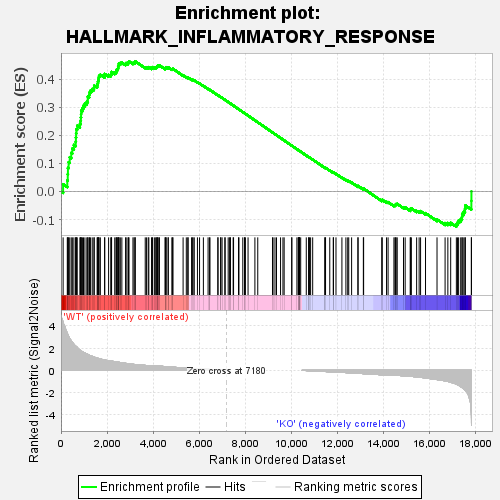

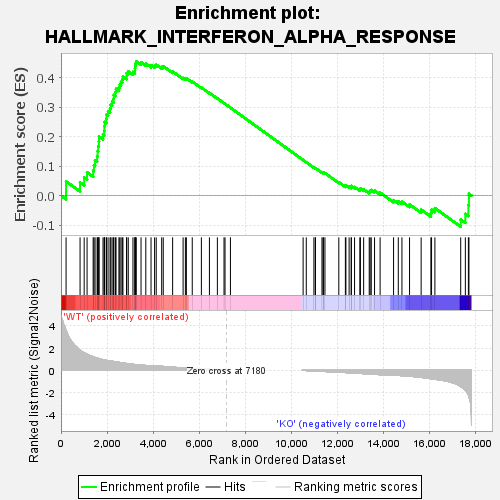


OVE4 parental vs p53R175H:


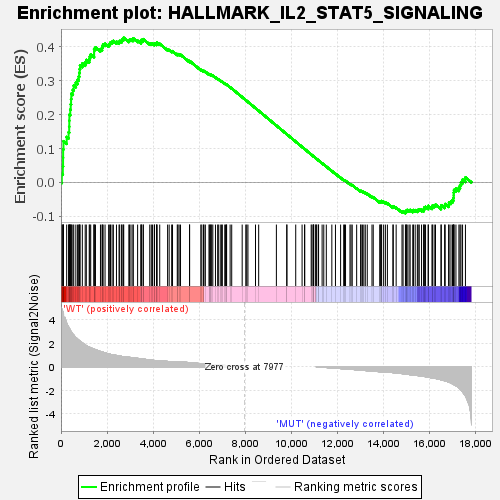

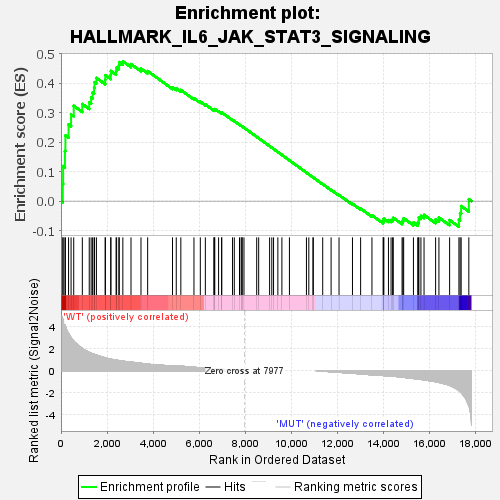

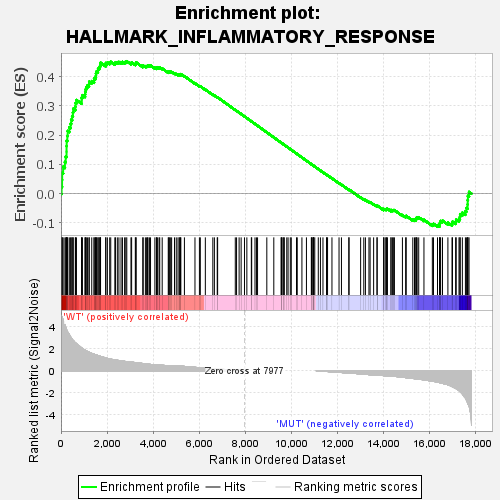

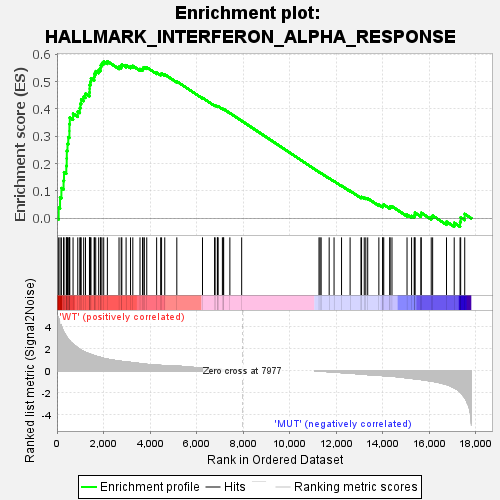

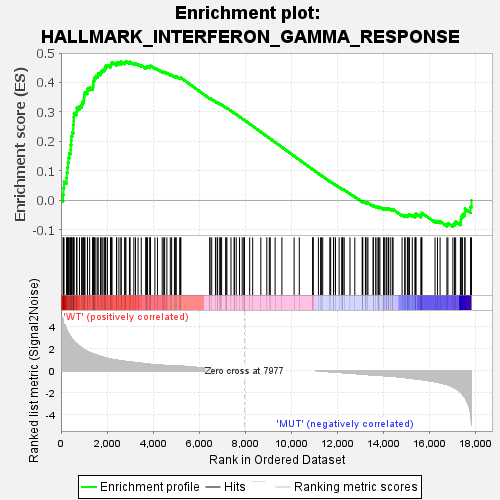

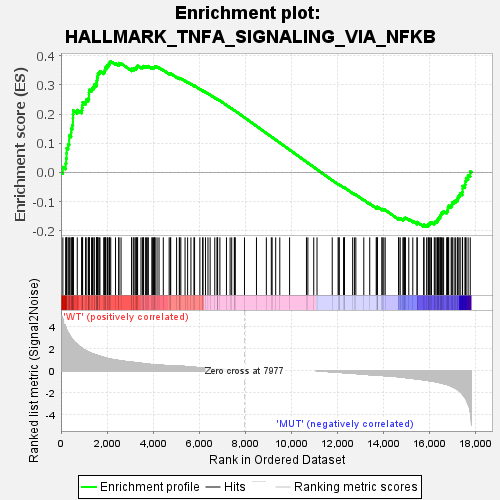


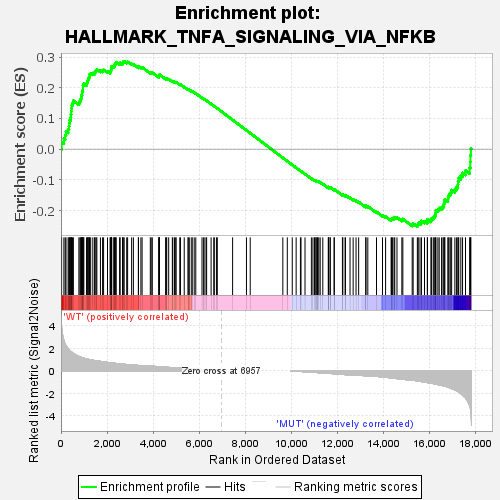

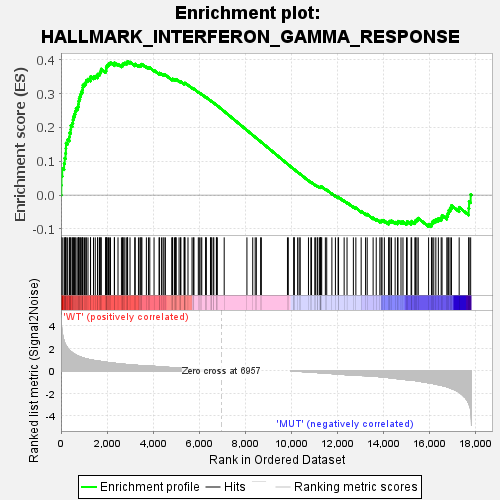

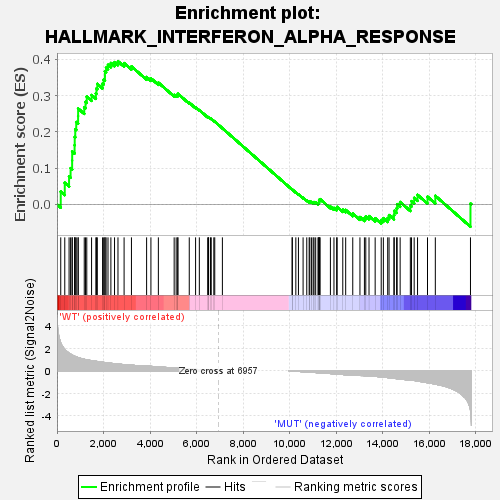

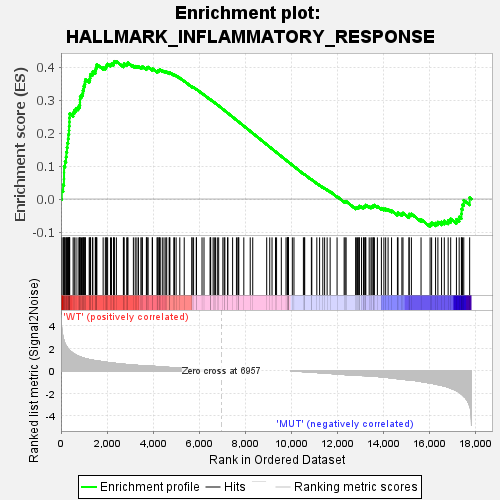

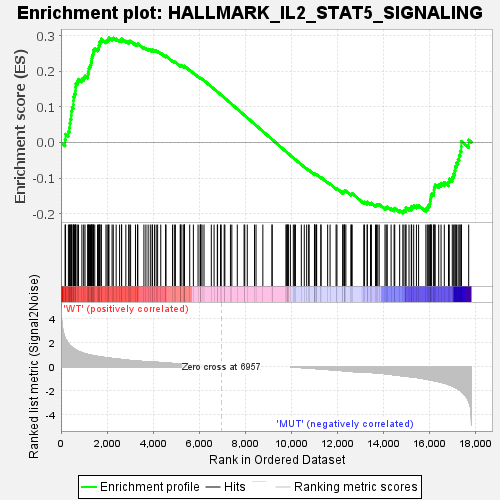

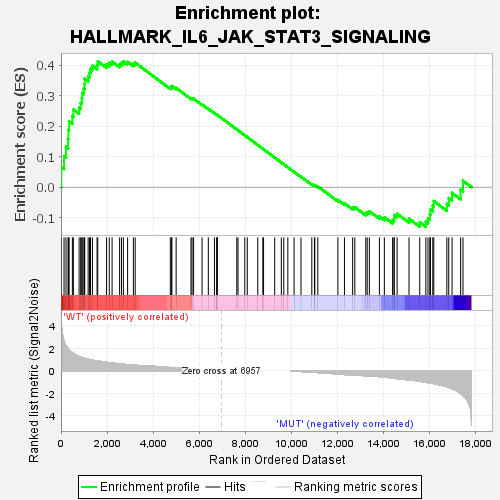


OVE4 parental vs p53R175H:

**Supplemental Figure S3:** Immune-related hallmark enrichment plots for the parental vs *Trp53*ko and parental vs p53^R175H^ comparisons (FDR < 0.25).


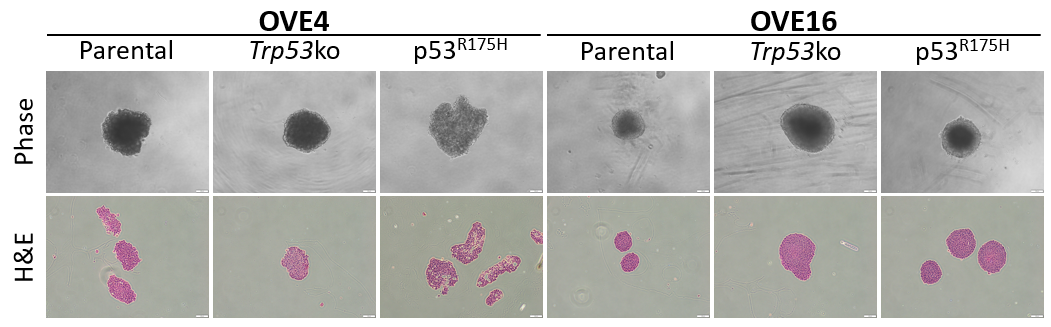


**Supplemental Figure S4:** Phase-contrast images and H&E sections of single OVE spheroids. Scale bars represent 100 µm.


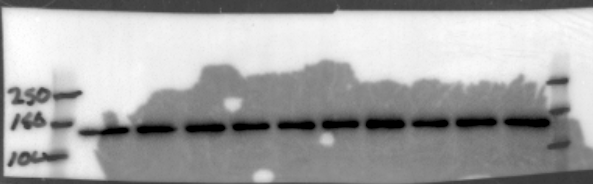


OVE16

OVE4

OVE4

OVE16


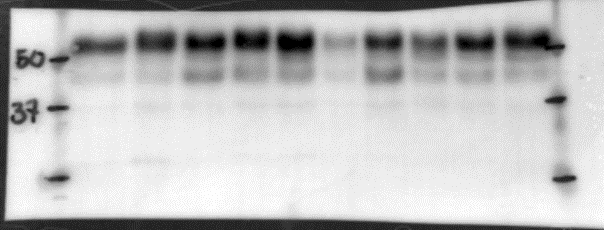


Figure 1A: Vinculin for Pax8

Figure 1A: Pax8


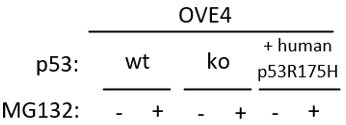

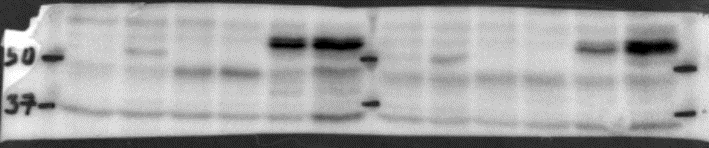

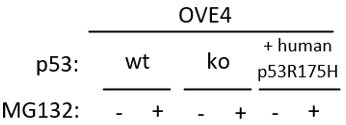


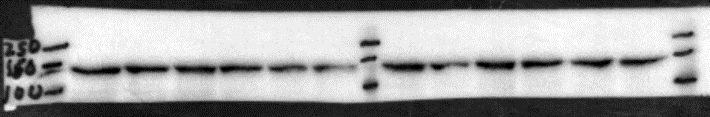


Figure 1B: Vinculin for p53

Figure 1B: p53


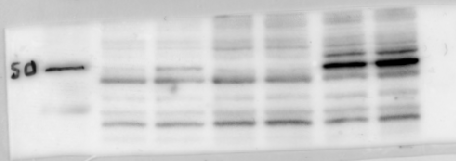

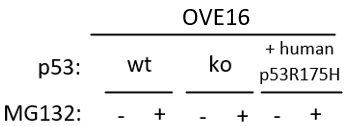


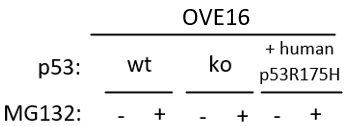

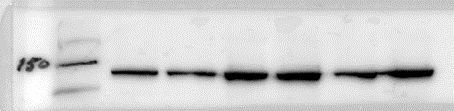


Figure 1C: Vinculin for p53

Figure 1C: p53

OVE4-p53^R175H^

OVE16

OVE4-p53^R175H^

OVE4-*Trp53*ko

OVE4

OVE16-p53^R175H^

OVE16-*Trp53*ko

OVE4

OVE4-*Trp53*ko

OVE4-p53^R175H^

OVE16

OVE16-*Trp53*ko

OVE16-p53^R175H^

OVE4

OVE16-p53^R175H^

OVE16-*Trp53*ko

OVE16

OVE4-*Trp53*ko


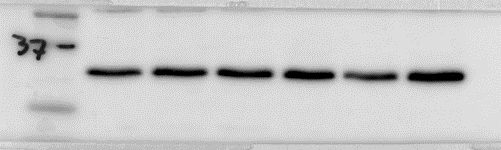

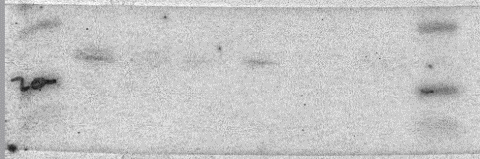

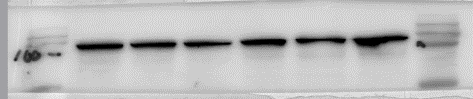

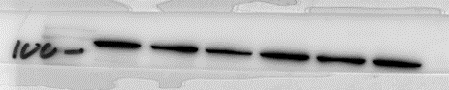

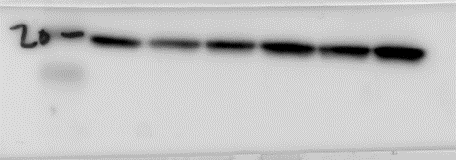


Figure 5A: Bcl-xl

Figure 5A: Bax

Figure 5A: Puma

OVE4

OVE4-*Trp53*ko

OVE4-p53^R175H^

OVE16

OVE16-*Trp53*ko

OVE16-p53^R175H^

OVE4

OVE4-*Trp53*ko

OVE4-p53^R175H^

OVE16

OVE16-*Trp53*ko

OVE16-p53^R175H^

OVE4

OVE4-*Trp53*ko

OVE4-p53^R175H^

OVE16

OVE16-*Trp53*ko

OVE16-p53^R175H^


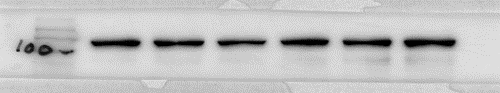


Figure 5A: Vinculin for Bax

Figure 5A: Vinculin for Bcl-xl

Figure 5A: Vinculin for Puma

OVE4-*Trp53*ko

OVE4-p53^R175H^

OVE16

OVE16-*Trp53*ko

OVE16-p53^R175H^

OVE4

OVE4

OVE4-p53^R175H^

OVE16

OVE16-p53^R175H^

OVE4-*Trp53*ko

OVE16-*Trp53*ko


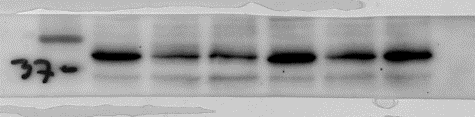

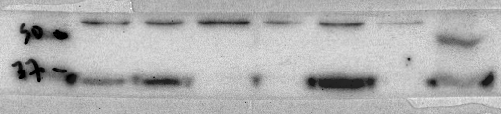


Figure 5C: Trailr2

Figure 5C: Trail

OVE4-*Trp53*ko

OVE4-p53^R175H^

OVE16

OVE16-*Trp53*ko

OVE16-p53^R175H^

OVE4

OVE4-p53^R175H^

OVE16

OVE16-p53^R175H^

OVE4-*Trp53*ko

OVE16-*Trp53*ko

OVE4


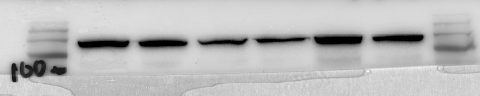

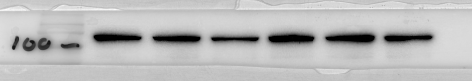


Figure 5C: Vinculin for Trail

Figure 5C: Vinculin for Trailr2

OVE16-p53^R175H^

OVE16-*Trp53*ko

OVE16

OVE4-p53^R175H^

OVE4-*Trp53*ko

OVE4

OVE16-p53^R175H^

OVE16-*Trp53*ko

OVE16

OVE4-p53^R175H^

OVE4-*Trp53*ko

OVE4


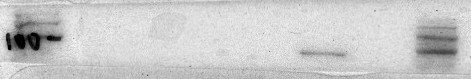


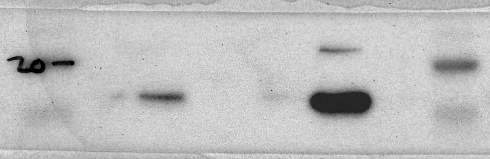


Figure 6A: Cleaved Parp

Figure 6A: Cleaved Caspase-3

OVE16-p53^R175H^

OVE16-*Trp53*ko

OVE16

OVE4-p53^R175H^

OVE4-*Trp53*ko

OVE4

OVE4

OVE4-*Trp53*ko

OVE4-p53^R175H^

OVE16

OVE16-*Trp53*ko

OVE16-p53^R175H^


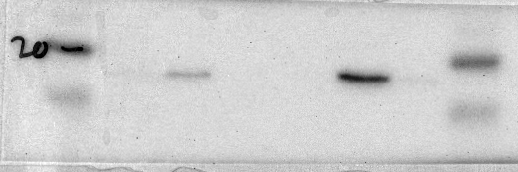


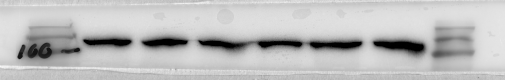


Figure 6A: Vinculin for cleaved Caspase-3

Figure 6A: Cleaved Caspase-7

OVE4-p53^R175H^

OVE16-*Trp53*ko

OVE16-p53^R175H^

OVE4

OVE4-*Trp53*ko

OVE16


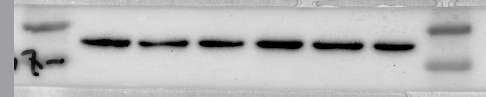


Figure 6A: Actin for cleaved Parp, cleaved Caspase-7

**Supplemental Figure S5:** Uncropped western blot membranes.

| **Gene** | **Primer Direction** | **Sequence (5’ to 3’)** |
| --- | --- | --- |
| *Cdkn1a* | F | TCGCTGTCTTGCACTCTGGTGT |
| *Cdkn1a* | R | CCAATCTGCGCTTGGAGTGATAG |
| *Slc20a1* | F | GCAAATGGGCAGAAGGGTGTCA |
| *Slc20a1* | R | CTTACGGAGGATGAACGCACGA |
| *Timp3* | F | AGGATGCCTTCTGCAACTCCGA |
| *Timp3* | R | GTGTAGACCAGAGTGCCAAAGG |
| *Tnfsf10* | F | GGAAGACCTCAGAAAGTGGCAG |
| *Tnfsf10* | R | TTTCCGAGAGGACTCCCAGGAT |
| *18s rRNA* | F | CCATCCAATCGGTAGTAGCG |
| *18s rRNA* | R | GTAACCCGTTGAACCCCATT |

**Supplementary Table S1:** Primers used for RT-qPCR validation
